# A Narrative Review of Old and Emerging Treatment Modalities for Substance Use Disorders

**DOI:** 10.7759/cureus.75395

**Published:** 2024-12-09

**Authors:** Tamanna Ahluwalia, Xiwen Xu, Andrew Nelson, Ishan Gujral, Jasmine Okafor, Michael R Hubbard

**Affiliations:** 1 School of Osteopathic Medicine in Arizona, A.T. Still University, Mesa, USA; 2 Osteopathic Medicine/Internal Medicine, A.T. Still University, Mesa, USA

**Keywords:** alcohol use disorder, behavioral therapy, contingency-management, medication assisted therapy, opioid use disorders, substance use disorder

## Abstract

Substance use disorders (SUDs) represent one of the leading causes of preventable death in the setting of overdose and comorbidities leading to mortality. A multi-database literature search limited to peer-reviewed articles within the last 10 years was conducted to compare treatment modalities used to treat SUDs. This literature review aims to provide a concise yet comprehensive summary of the various treatment modalities that exist to address these disorders in clinical practice. Healthcare professionals in multiple disciplines are encouraged to continue exploring the modalities outlined in this review to competently and holistically address the various factors that contribute to these disorders. This literature review also serves as an important reminder of the ongoing evolution and range of treatments for SUDs.

## Introduction and background

Substance use disorders (SUDs) encompass a wide range of symptoms related to the continued use of a substance despite its negative effects. A neurobiological illness such as any other medical condition, addiction affects various brain pathways involved in reward, motivation, and inhibitory control. The most recent Diagnostic and Statistical Manual of Mental Disorders (DSM) clarifies SUD by 11 criteria that fall under four subgroups: impaired control, physical dependence, social issues, and risky use [1]. The severity of substance misuse may be classified by the number of criteria an individual meets, allowing clinicians to guide their treatment. One out of the 11 symptoms may point to an at-risk patient; two to three signify mild SUD; four to five indicate moderate SUD; and six or more symptoms represent severe SUD [1]. SUD is an overarching term comprising specific substances within it, including opioids, alcohol, cannabis, tobacco, and many more. Similar to other medical conditions treated in primary care and hospital settings, risk factors for developing SUD include, but are not limited to, genetic polymorphisms, environmental factors (such as early exposure to alcohol or drugs and childhood trauma), and psychiatric comorbidities (such as bipolar disorder, depression, and anxiety). According to He et al., children with high adverse childhood experiences (ACEs) have been shown to have a greater association with drug addiction [2].

In 2022, 48.7 million people aged 12 or older (or 17.3%) had a SUD in the past year, including 29.5 million who had an alcohol use disorder (AUD), 27.2 million who had a drug use disorder (DUD), and 8.0 million people who had both an AUD and a DUD [3]. SUDs remain a major cause of preventable death worldwide. While screening and prevention strategies are commonplace for other potentially fatal medical diseases, such as hypertension and diabetes, these modalities are used only sporadically in the primary care setting for individuals with SUD. Various screening tools, such as the Car, Relax, Alone, Forget, Friends, Trouble (CRAFFT) questionnaire for adolescents and the Cut down, Annoyed, Guilty, and Eye-opener (CAGE) questionnaire for adults, have been shown to reduce the quantity of alcohol that patients use [4]. When managing pain, physicians should escalate from non-opioid medications to opioids in a stepwise manner. Before initiating opioid medications, evaluating patients for risk factors is crucial. Physicians should also engage in vigilant and consistent use of prescription drug monitoring programs (PDMPs), as they have been shown to reduce “doctor shopping” and reduce prescription drug misuse [5]. To be effective, primary prevention interventions must address psychosocial and environmental risk factors, as well as protective factors at the individual, interpersonal, and macroscopic levels, to prevent the onset of SUD [6]. Moreover, a multidisciplinary approach is helpful in tackling the complex issues that patients with SUD face.

One of the primary issues that patients with SUD struggle with is the return to the use of a previous drug that has led to addictive traits. A personalized and culturally sensitive treatment approach must be taken by all healthcare professionals involved in the care of SUD patients to optimize their quality of life. A key component missing from the majority of the existing literature is the lack of integration between multidisciplinary treatment modalities, with papers often focusing on either medication-assisted treatment or cognitive behavioral therapy. This literature review aims to bridge this gap by comprehensively summarizing the various modalities that exist to address SUD, ranging from medication for opioid use disorder to neuromodulation techniques. Since SUD is a broad condition encompassing multiple substances, we will also delve into specific (but not all) substances to illustrate the effectiveness of a treatment modality. We also explore novel treatment strategies being researched and various countries’ approaches to treating SUDs. In doing so, we hope that healthcare professionals will have a better understanding of the options available for the treatment of SUDs.

Methods

A multi-database literature search was conducted to compare treatment modalities used to treat substance use disorder. Five databases were searched for peer-reviewed studies. The following databases were searched: PubMed (NIH), Google Scholar, Nursing and Allied Health (Gale), PsychINFO (EBSCO), and Cumulative Index of Nursing and Allied Health Literature (CINAHL, EBSCO). The search criteria used included the keywords “substance use disorder”, “substance related disorders”, “behavioral therapy”, “medication therapy”, “therapy”, and “treatment”. All searches that were part of the study were limited to a publication date within the last 10 years. The resulting studies were organized using a spreadsheet and divided into four groups based on the treatment modality used to treat SUD. The four groups were as follows: medication-assisted treatment, therapy-based treatment, exercise-based treatment, and novel/non-Western/upcoming treatments. The studies were reviewed and summarized on a spreadsheet. Subsequently, the results and effectiveness of these treatment modalities were compared in regard to their results and effectiveness in reducing the return to use of the substance in question.

## Review

Medications for opioid use disorder (MOUD) in SUD management: a comprehensive overview

The effectiveness of MOUD in addressing opioid use disorder (OUD) has been well established across multiple studies. MOUD significantly contributes to improving treatment outcomes and fostering sustained engagement with healthcare services among individuals experiencing OUD. 

Effectiveness Across Various Substances and Treatment Modalities

Research highlights the efficacy of MOUD in reducing opioid cravings and withdrawal symptoms, with medications such as buprenorphine and methadone significantly improving treatment retention rates [7]. An overview of the characteristics of MOUD, including their mechanism of action, uses, advantages, and disadvantages is shown in Table 1 [7]. Studies have also indicated an association between MOUD after nonfatal opioid overdose and mortality. One retrospective cohort study between 2012 and 2014 found buprenorphine and methadone prescribed following an overdose led to a reduction in all-cause mortality and deaths associated with opioid use [8]. Their findings help support the importance of MOUD in addressing both opioid and non-opioid-related deaths from those with OUD. Recent Medicare data from 2008-2016 compared data following a nonfatal opioid overdose between buprenorphine treatment and standard psychosocial treatment for OUD. It found patients given buprenorphine to have a 62% reduction in the risk of opioid overdose compared to no significant reduction in the psychosocial group. However, it was noted that less than one in 20 patients were found to be on buprenorphine in the following year, highlighting a potential gap in adequate MOUD utilization [9]. Another study highlighting potential gaps in MOUD for vulnerable populations showed that states that covered MOUD as part of Medicaid had 45% of their participants on MOUD compared to 31% in states that had block grant coverage and only 17% in states with no coverage [10].

**Table 1:** Characteristics of medications used in the treatment of OUD Reference number [7]. Appropriate permissions from original authors have been sought and obtained. OUD: Opioid use disorder.

Medication	Methadone	Buprenorphine	Naltrexone
Brand Names	Dolophine, Methadose	Subutex, Suboxone, Zubsolv	Deprade, Vivitrol, ReVia
Class	Agonist (fully activates opioid receptors)	Partial agonist (activates opioid receptors, but produces a diminished response even with full occupancy)	Antagonist (blocks the opioid receptors and interferes with the rewarding and analgesic effects of opioids)
Use and Effects	Taken once per day orally to reduce opioid cravings and withdrawal symptoms	Taken orally or sublingually (usually once a day) to relieve opioid cravings and withdrawal symptoms	Taken orally or by injection to diminish the reinforcing efects of opioids (potentially extinguishing the association between conditioned stimuli and opioid use)
Advantages	High strength and efficacy as long as oral dosing (which slows brain uptake and reduces euphoria) is adhered to; excellent option for patients who have no response to other medications	Eligible to be prescribed by certified physicians, which eliminates the need to visit specialized treatment clinics and thus widens availability	Not addictive or sedating and does not result in physical dependence; a recently approved depot injection formulation, Vivitrol, eliminates need for daily dosing
Disadvantages	Mostly available through approved outpatient treatment programs, which patients must visit daily	Subutex has measurable abuse liability; Suboxone diminishes this risk by including naloxone, an antagonist that induces withdrawal if the drug is injected	Poor patient compliance (but Vivitrol should improve compliance); initiation requires attaining prolonged (e.g., seven days) abstinence, during which withdrawal, relapse, and early dropout may occur

The Case for Buprenorphine and Methadone as First-Line Therapy for MOUD

Comparative analyses between MOUD and therapy-centered interventions showed that the two used in conjunction may have potentiating effects. Studies further emphasize the importance of integrating MOUD with motivational interviewing (MI) to address both physiological and psychological aspects of addiction [11,12]. Despite this evidence previously highlighting the importance of cognitive behavioral therapy (CBT) used concomitantly with MOUD, the evidence over recent years seems to have shifted. 

The aforementioned studies discussed that simply receiving MOUD following a nonfatal opioid overdose was associated with a significant reduction in all-cause and opioid-related mortality [8]. In light of these findings, a retrospective comparative study in 2020 concluded that medications such as buprenorphine or methadone were associated with lower mortality rates, greater treatment engagement, reduced healthcare utilization, and increased sustained abstinence than non-MOUD outpatient treatment, residential treatment, or detoxification alone [13]. Based on this, it was declared that either buprenorphine or methadone should be utilized as first-line management for MOUD, highlighting the need for expanded access to evidence-based MOUD programs. 

While MOUD is the gold standard of treatment, an additional retrospective cohort study was conducted in Vancouver, British Columbia that evaluated the provinces’ COVID-19 pandemic policies. These were aimed at reducing unnecessary exposure to COVID-19 and simultaneously addressing issues related to overdose, withdrawal, and exposure to the harms of interfacing with an unsafe drug supply. The policies in place were designed to implement a “safe supply” of medicines such as opioids, stimulants, benzodiazepines, and alcohol withdrawal management medications. Their findings were associated with reduced overdose deaths and all-cause mortality among people with OUD, leading them to conclude that pharmaceutical alternatives to the drug supply might provide another way to reduce mortality for people with OUD [14]. These studies provide valuable insights into the effectiveness of MOUD, particularly in reducing mortality among individuals with OUD. They suggested that MOUD alone can be a highly effective intervention and may offer advantages in certain situations compared to combination therapy with CBT.

Addressing Adherence and Treatment Engagement

Retention to methadone and buprenorphine treatment is associated with substantial reductions in the risk for all-cause and overdose mortality in individuals dependent on opioids. According to a systematic review in 2017, the induction phase of methadone treatment and the time immediately after leaving treatment with both drugs are precarious periods of increased mortality risk, which should be addressed by both public health and clinical strategies to mitigate such risk [15].

Considerations for Implementation and Barriers

Despite its well-studied effectiveness, significant barriers to MOUD administration remain. Several studies help identify challenges related to provider training, limited access to medications, and stigma as key barriers to MOUD utilization [16,17]. Addressing these barriers is key to helping maximize the potential of MOUD. Various studies from the patient’s perspective have found that stigma was the primary barrier to the use of MOUD, while providers cited logistical and knowledge gaps as the biggest reason for their lack of MOUD utilization [18]. Steps in addressing barriers to prescribing MOUD should thus center around patient interactions and address the stigma they experience when interfacing with the medical system. Several studies have demonstrated effective strategies for eliminating other barriers. While stigma remains a challenge, there are major barriers to receiving MOUD for those living in rural communities. Prior to the elimination of the DEA X-waiver in 2018, it was found that more than 60% of rural counties in the U.S. lacked a prescriber with a waiver to prescribe buprenorphine [17]. With the elimination of the X-waiver in 2023, further research is needed to evaluate whether this has led to an increase in access for rural communities. Other studies have identified a variety of reasons why MOUD is not fully implemented, the scope of which is beyond this review. Strategies that were identified across the board to reduce barriers to MOUD included policy changes, workforce development, the integration of MOUD into primary care settings, and patient engagement initiatives [16].

Future Directions and Recommendations

In identifying the leading barriers and stigma that continue to limit the full utilization of MOUD in healthcare settings, recommendations for how to best move forward are discussed. It seems that a comprehensive integration of MOUD into healthcare settings, along with ongoing provider education, can be an effective strategy. Incorporating MOUD into primary care is an effective way to help expand access and improve overall treatment outcomes [19,20]. Many changes in telehealth prescribing, as seen during the COVID-19 pandemic, are still being implemented today. These changes have led to increased ease of access, particularly for patients struggling with transportation and the host of challenges that may be in place for people experiencing OUD.

In summary, these findings help identify barriers such as stigma as detrimental to patient care and the ways MOUD can be used as first-line treatment. Addressing barriers and enhancing comprehensive care models are imperative to harness the full potential of MOUD. 

The role of behavioral treatment in SUD management

Non-abstinence Treatment as an Option for Alcohol Use Disorder (AUD)

Although achieving abstinence is known to be the ideal goal for treating those with SUD, many patients do not wish to completely eliminate substance use. This is even more common among those who do not seek treatment. Non-abstinence treatment was first introduced in the 1970s in a study that utilized controlled drinking to treat inpatient individuals with AUD, and the results were better than those of the standard protocol. After a year of completing treatment, more than a quarter of the patients maintained controlled drinking. Studies have extrapolated this information and performed similar experiments to examine the benefits of non-abstinence treatment on those with drug use disorder (DUD), as there is a lack of research on this topic compared to that on those with AUD. It is believed that non-abstinence treatment could be a potentially greater appeal to patients with SUD to seek treatment earlier before the disorder leads to life-threatening consequences. As discussed by Paquette et al. (2021), a recent study showed that those who seek treatment for SUD have more harmful effects than those who do not seek treatment [21]. People are more likely to wait for treatment until their symptoms increase in severity and repercussions result, thus demonstrating a need to seek help earlier. Non-abstinence treatment not only includes lowering and controlling substance use but also focuses on improving quality of life, improving health, and maintaining connections with supportive services. These goals are also aligned with those of most adults diagnosed with SUD who do not desire complete abstinence, especially high-risk individuals. Non-abstinence psychotherapies offer strategies to attain such goals, but many studies have focused on AUD, with limited research on DUD.

Harm reduction psychotherapy is a model that centers on the idea of non-abstinence and involves MI and CBT to increase encouragement, provide skills to control substance use, explore reasons for addiction, and build trust in therapy. This approach has been shown to be extremely effective in reducing use for individuals with AUD, particularly for those in earlier stages. There is also additional support from peers in harm reduction therapy for groups, which may consist of patients with different goals. An example studied by Schippers and Nelissen (2009) included an outpatient SUD treatment center in Europe where harm reduction psychotherapy has been implemented in community-based SUD treatment [22]. Methods that directed this treatment included MI and CBT. Paquette et al. (2021) wrote that “participants with controlled use goals in this center are typically able to achieve less problematic (38%) or non-problematic (32%) use, while a minority achieve abstinence with (8%) or without (6%) incidental relapse (outcomes were not separately assessed for those with AUD vs. DUD)” [21]. Although there is scarce research on such treatments in those with DUD, the core approach is similar to that used for those with AUD in the process of achieving non-abstinence goals.

Motivational Interviewing 

MI was originally created for individuals with AUD, but it has been used for individuals with SUD, including cannabis and other drug use. Studies have shown that there is no significant benefit of MI over other treatments for SUD. Paquette et al. (2021) demonstrated that the remaining studies that showed the maximum benefit of MI were most prominent in its purpose as an adjunct or as an initiating step for abstinence-centered treatment [21]. More research in this area would likely benefit this model’s implications in harm reduction therapy.

Cognitive Behavioral Therapy 

Another model important for treatment outcomes is CBT. A study conducted in Western Sweden by Bador and Kerekes (2019) showed the importance of integrated intensive CBT treatment for 35 outpatients suffering from substance use and mental health disorders [23]. The most commonly associated mental illnesses were anxiety and depression, which were notably decreased at the end of the study. Instead of using previous approaches to treat different diagnoses in different facilities or delegating the precedence of SUD in patients with coexisting mental illnesses, this integrative intensive CBT treatment attends to all of the associated illnesses and problems. This emphasizes the multiple factors that can contribute to SUD, and vice versa. CBT was the primary model for tapping into patients’ thought processes in an attempt to change their behavior and facilitate positive emotions. Interventions by Bador and Kerekes (2019) included “psychoeducation, cognitive processing, modulation, problem-solving exercises, affect regulation, exposure and response prevention, behavior experiments and activation, mindfulness, skills training, auricular acupuncture (NADA), and home assignments” [23]. Assessment scales were examined, and there were found to be significant improvements in anxiety, depression, and hopelessness, which are risk factors for substance use. Bador and Kerekes (2019) observed improvements in self-esteem and the experience of meaningfulness, which are motivation factors for people to continue to stay away from substances [23]. In addition to preventing relapse through increasing motivation factors, this treatment also served to help patients with mood disorders-which may have propelled them to reach for substances initially. This integrated intensive CBT treatment aims to achieve dual effects, and it has provided immensely positive results that should be further exemplified in further studies.

In summary, traditional CBT therapy has been widely studied and shown to be effective in treating AUD and other SUDs. Its effectiveness with other models, such as motivational interviewing and contingency management, increases the appeal of treatments to patients and helps them maintain progress. Although this approach is prevalently used for abstinence-based treatment, non-abstinence treatment essentially shares the same concept of alleviating substance use and increasing patient well-being. Perhaps non-abstinence treatment is the key to initiation and efficiency, with the addition of CBT therapy and other strategies discussed to treat the multifaceted stages of SUD.

Exercise-based treatment modalities and their biopsychosocial benefits in the treatment of SUD

The standard of care as it relates to SUD has typically centered around medication and behavioral therapies, as outlined thus far. While these treatment modalities are surely effective, research continues to delve into additional and alternative methods that may enhance patient care and abstinence in those with SUD. Although the success of medication-based therapy for SUD has been well documented, the utility of exercise-based treatment (EBT) as an adjunct therapy for SUD may improve patient outcomes. A study performed by Abrantes and Blevins (2019) revealed the biochemical mechanisms behind exercise, particularly aerobic exercise, and how this may be beneficial in treating individuals with SUD [24]. Aerobic exercise, like other forms of exercise, naturally stimulates the production of endorphins as well as endogenous opiates. These endogenous opiates, similar to many drugs of abuse, thereby activate the dopaminergic signaling system in the brain, improving mood and energy and potentially producing a euphoric feeling. This study suggested that exercise has the potential to normalize the disrupted dopaminergic signaling system in individuals with SUD and is therefore a valuable adjunct treatment to medication and behavioral therapies.

The impact of physical exercise on SUDs was analyzed in a meta-analysis by Wang et al. (2020) in which the results focused on four primary outcomes related to SUD: abstinence rate, symptoms of withdrawal, anxiety, and depression [25]. Wang et al. reported that "physical exercise can more ease the depression symptoms in alcohol and illicit drug abusers than nicotine abusers, and more improve the abstinence rate on illicit drug abusers than the others” [25]. Physical exercise was, therefore, not only beneficial in reducing relapse but also had significant psychological impacts on mental well-being by easing withdrawal symptoms, anxiety, and depression. The researchers further broadened the scope of the study by including mind-body exercises such as Qigong, Tai Chi Quan, and Yoga and reported that these modalities have treatment effects similar to those of aerobic exercise [25].

Exercise also has the ability to be valuable in the treatment of AUD. In studies conducted by Brown et al., it was found that over the course of 12 weeks, participants who engaged in regular exercise in an outpatient alcohol rehabilitation program were found to have significant reductions in drinking outcomes compared to those who did not adhere to the exercise intervention [26]. Furthermore, in a cross-sectional analysis by Weinstock et al. (2008), this impact was further analyzed, and it was found that the exercise groups had longer periods of abstinence from alcohol as well [27].

While studies such as those by Abrantes and Blevins (2019) [24], Wang et al. (2020) [25], and Brown et al. (2010)[26] found significant benefits of EBT in treating SUD, other studies such as those performed by Alessi et al. (2020) highlight that the effectiveness of EBT in treating SUD is an area of research that requires further investigation [28]. The study compared exercise-reinforced contingency management (CM-Exercise) to general contingency management (CM-General) in the treatment of SUD. A total of 120 participants were included in the study. The CM-General intervention group was based on standard outpatient SUD treatment, including medication and behavioral therapies, whereas the CM-Exercise intervention group focused on adding physical activity to the aforementioned therapies. The study revealed that the CM-Exercise group did not have an advantage compared to the CM-General group in regard to decreasing substance use. However, the study did not differentiate outcomes between a CM-exercise group and a group with no CM whatsoever. Therefore, this study may highlight that CM interventions are potentially effective tools for treating SUD rather than focusing on the benefit of EBT compared to standard treatment for SUD without CM.

Limitations of EBT and Future Directions

The use of exercise in treating SUD is an exciting potential adjunct therapy to the mainstays of treatment, including medication and behavioral therapy. However, it remains relatively new within the literature, and few meta-analyses have been performed directly addressing the impact of physical exercise on SUD. Thus, it is important for providers to be cognizant of the potential drawbacks of EBT. In regard to patient adherence, EBT poses numerous confounding factors, including issues such as inadequate transportation or financial limitations. Abrantes and Blevins reported that drop-out rates from EBT groups were greater than those from medication and behavioral treatments, likely due to the aforementioned factors, as well as due to the comorbid cognitive and mental health difficulties those with SUD often face [24]. Therefore, research aimed at understanding exercise regimens least likely to promote attrition and decrease failure rates in those suffering from SUD would likely improve patient abstinence. While current research and literature continue to analyze the effectiveness of EBT in treating SUD, studies including those by Alessi et al. (2020) emphasize that reinforcing exercise has numerous psychosocial benefits [28]. These include promoting self-efficacy and positive coping strategies, both of which are important factors in the biopsychosocial recovery that many patients with SUD face. Stoutenberg et al. (2016) understood self-efficacy as related to SUD as “the extent to which the addicted individual views themselves as being able to cope with a difficult situation without relying on alcohol or other drugs” [29]. Stoutenberg et al. (2016) reported that exercise regimens are associated with enhanced self-efficacy and capability, which are important characteristics of individuals seeking treatment for SUD [29]. The study noted that research measuring improvements in self-efficacy secondary to exercise in those specifically with SUD has not been conducted. Studying this impact would likely be beneficial in future studies and research to understand the true scope of the psychological benefit of exercise in treating SUD. However, the measurability of criteria such as self-efficacy remains a challenge and has not been fully investigated in studies and meta-analyses. Cabè et al. (2021) recognized these challenges and argued that future randomized controlled trials (RCTs) should include and specifically measure neuropsychological craving and impulsivity assessments in those with SUD [30]. The impact of physical exercise on these criteria would be an interesting future area of research, particularly given the effects of exercise on the dopaminergic reward signaling pathway in the brain.

Novel and non-western treatment modalities for SUD

As innovative research continues on the topic of SUDs, we find that there are many treatment modalities that do not fall under the umbrella of standard care. While conventional treatments are often the mainstay of management, there is a growing presence of more novel and non-Western ideas. Current treatments tend to focus on different modes of psychotherapy and pharmacotherapy, with the ultimate goal of reduced or complete abstinence during consumption. While the use of novel treatments is growing, the results of these interventions can be inconsistent and complex [31]. However, with the global issue that SUD poses, larger and more original studies are being pushed and conducted both within and outside the United States to fully explore the possibility of utilizing these different ideas. As these designs are being implemented and tested it is now more important that we analyze what has been done to potentially facilitate new models of research for SUDs. We are seeing modified strategies for our therapeutic interventions, incorporation of technology, and new pharmacological approaches.

Therapeutic Interventions

CBT is the foundation of modern-day therapeutic intervention for SUD, as described above. However, novel therapeutic approaches have now begun to be developed. Mindfulness with roots in Eastern tradition, which has more prominently been used for psychiatric disorders due to its effects on mood and anxiety, has now been applied and tailored in a variety of ways to treat SUD [31]. There is a high comorbidity prevalence of those with SUDs and mood disorders such as anxiety and depression. Lai et al. (2015) conducted a systematic review and meta-analysis to determine the strength of the relationships among SUD, mood, and anxiety disorders. The review confirmed the strong correlation, particularly with depression, followed by anxiety [32]. This strong association can affect the severity and outcomes of SUDs. In a preliminary randomized controlled study performed in Brazil, the treatment group (experimental group n=22), which involved mindfulness in addition to the usual treatment, had fewer symptoms of depression and anxiety than did the treatment as usual group (control group n=22). There was no significant effect on SUDs [33]. Larger studies are needed, but the use of mindfulness adjuvants has shown promising results.

Acceptance and Commitment Therapy (ACT) is an up-and-coming behavioral therapy that focuses on accepting, rather than avoiding, challenges in one's life. When applied to SUD, individuals are encouraged to accept the urges and symptoms pertaining to substance use and then use value-based interventions to reduce said urges. Svanberg et al. 2017 conducted a literature review of studies utilizing ACT. A web-based ACT study on smoking cessation was performed in both 2013 and 2015, with results from both showing a higher cessation rate compared to the control [34]. The use of ACT in an RCT of 31 incarcerated women with SUD showed a 27.8% reduction in substance use at the end of the study, with an increase to 43.8% at the end of the six-month follow-up [34]. Overall, ACT has shown encouraging results in reducing substance use at the end of treatment and within a follow-up period.

Technology (Digital Age)

With the growing use of this technology, it is only natural to incorporate these newer advances in the treatment of SUD. Previous studies have shown the efficacy of computer-based cognitive behavior therapy (CBT4CBT) as an additional modality to the standard treatment of care [35]. Kiluk et al. (2018) explored the use of CBT4CBT as a stand-alone treatment [35]. A total of 137 individuals were randomized into three treatment groups: standard treatment at the clinic with either weekly group or individualized therapy, clinician-delivered CBT with 12 individual sessions of manual-guided CBT delivered by clinicians, and the final group in which participants completed one CBT4CBT module a week with a weekly brief in-person clinical monitoring session [35]. Compared with treatment as usual, CBT4CBT and clinician-delivered CBT significantly reduced the frequency of substance use. A six-month follow-up revealed continued benefits of CBT4CBT over standard treatment but not clinician-delivered CBT. The secondary outcomes of the study showed that CBT4CBT had the greatest retention, demonstrated the best learning of cognitive and behavioral material, and had the greatest satisfaction with treatment among participants [35]. While clinician-delivered CBT indicated high rates of effectiveness, it was associated with an increased dropout rate and decreased effects as follow-up continued.

Another study by DeFulio et al. (2022) investigated the use of smartphone-based contingency management with the primary outcome measurement of medication adherence, specifically for buprenorphine [36]. Smartphone contingency management has been successful in promoting antiretroviral treatment in those living with Human Immunodeficiency Virus (HIV). Petry et al. (2011) define contingency management as a behavioral therapy that hones in on an individual being reinforced or rewarded for evidence of positive behavioral change [37]. It has been shown to be effective in treating SUD. The DeFulio study followed a group of 20 participants for 12 weeks, and each participant was given a smartphone app that provided contingency management with peer recovery coaching. Buprenorphine adherence was determined by Global Positioning System (GPS) monitoring of clinic medication visits and self-recorded videos. In addition, a salivary toxicology test was performed weekly to assess adherence. Throughout the treatment, 100% of participants were retained in the study, with 76% confirmed adherence to buprenorphine [36]. These results show the potential for smartphone technology to be used as a vehicle for SUD treatment.

Extensive research has been conducted regarding the underlying changes in brain activity in individuals with addiction and SUDs. Neuromodulation is a type of technology that acts directly upon circuits in the brain [31]. The use of noninvasive brain stimulation devices such as transcranial magnetic stimulation (TMS) and transcranial direct current stimulation (tDCS) within the context of SUD has been discussed. For example, tDCS applies a continuous weak electrical current that is attached directly to the scalp. Both neuromodulation techniques have been studied in populations with addiction, specifically directed toward the dorsolateral prefrontal cortex [31]. Song et al. conducted multiple meta-analyses of the effects of neuromodulation technology on substance and food addiction [38]. Neuromodulation of the dorsolateral prefrontal cortex significantly decreased cravings immediately afterward, and sustained reductions in consumption and craving were also observed [38]. Although these studies are still in the preliminary stages, the use of technology is very promising for the future treatment of SUDs.

Repurposing Pharmacotherapy

Pharmacotherapy has become increasingly popular for the treatment of SUDs. Although non-pharmacological treatments continue to be the desired course of action, there are benefits associated with the addition of traditional medications such as buprenorphine, naltrexone, and methadone. However, there is a new line of treatment that focuses on repurposing FDA-approved drug therapies as a way to identify alternative treatments for SUDs [38]. Psychiatric medications are often co-morbid, with the ability to treat both psychiatric and SUDs. An example is sertraline, a selective serotonin reuptake inhibitor that not only treats depression but also reduces alcohol consumption in individuals with AUD [38]. Patel et al. (2023) conducted a retrospective observational study using the TriNetX electronic medical records system to evaluate FDA-approved medications that have concurrent use in those diagnosed with OUD [39]. Of the 103 medications screened, 28 were selected. Buprenorphine and methadone were selected as positive controls due to their effectiveness in treating OUD. Of the 28 medications, eight in the absence of methadone and buprenorphine were found to have significant effects on increasing the proportion of OUD individuals in remission [39]. Some of these medications included prazosin, olanzapine, quetiapine, and propranolol. In addition, the study showed that among the 28 drugs selected in the presence of buprenorphine or methadone, there was a significant increase in OUD remission [39].

There have been new studies examining alternative medications as possible modes of treatment. A study conducted at the Tawasaki Center in Peru investigated the use of Amazonian medicine in conjunction with Western psychotherapy [40]. The duration was between three and twelve months, depending on the individual's needs. The Amazonian methods applied to the participants fell under three categories: 1) purging rituals where plants were used with emetic effects, 2) weeklong dietary forest retreats where participants are secluded and administered medicinal plants, and 3) a healing ceremony after the initial detoxification where specific plants are given (namely, the ayahuasca). These different settings helped provide a sense of understanding and resolution of emotional trauma [40]. In the Berlowitz study of 53 individuals, 36 completed the study, and the results showed a significant decrease in craving frequency, emotional distress, and overall quality of life. Although preliminary, these positive results encourage further investigation.

Cannabinoids have also become a topic of discussion among a multitude of different disease states. Specifically, cannabidiol (CBD), the least addictive part of cannabis, has been studied for its use in multiple SUDs. A double-blind RCT investigated the effectiveness of CBD (400 or 800 mg) in reducing cue-induced cravings and anxiety during drug abstinence [41]. These results were consistent with a reduction in cravings and anxiety after heroin cues [41]. Like cannabinoids, psychedelics have become increasingly popular for understanding different ways to apply these methods to the treatment of SUD. Sharma et al. (2023) conducted a systematic review of an RCT, four case series, and two before-and-after studies investigating the benefits of psychedelic treatments on SUD [42]. A total of 324 participants were studied, but the results were ultimately inconclusive. There were many limitations within the study, such as the lack of a well-established control group and significant loss to follow-up [42]. Therefore, the data were ultimately inconclusive. Improvements in mood, depression, and quality of life were reported for all psychedelic substances investigated [42]. Ultimately, many of these alternatives to traditional pharmacotherapy are in the very early stages of exploration and research. The current lack of established research leads to difficulty in fully understanding the role these substances can play in SUDs.

## Conclusions

SUDs represent a significant public health challenge, affecting millions of individuals worldwide and contributing to a substantial burden of preventable mortality. Despite our understanding of the neurobiological basis of addiction and the availability of effective screening and prevention strategies, SUDs continue to be under-diagnosed and under-treated in many healthcare settings. Our literature review aims to address this gap by providing an overview of the various modalities available for the treatment of SUDs, ranging from traditional pharmacotherapies to emerging neuromodulation techniques. By exploring novel treatment strategies and examining approaches from different countries, we aim to provide healthcare professionals across various disciplines with the knowledge and tools necessary to reduce preventable deaths and improve the quality of life for individuals with SUDs. In that manner, we believe that a culturally sensitive approach to treatment, informed by evidence-based practices and innovative research, can help reduce the negative impact for those living with substance use and alcohol use disorders.
